# The effect of Internet use on body weight in Chinese adolescents: Evidence from a nationally longitudinal survey

**DOI:** 10.1371/journal.pone.0311996

**Published:** 2024-12-16

**Authors:** Junqi Ma, Li Sheng

**Affiliations:** 1 School of Public Administration, Chongqing Technology and Business University, Chongqing, China; 2 School of Foreign Languages, Chongqing Technology and Business University, Chongqing, China; King Faisal University, SAUDI ARABIA

## Abstract

The prevalence of overweight and obesity among adolescents has been increasing steadily. This study aims to investigates the causal effect and underlying mechanisms between Internet use and body weight among Chinese adolescents. Using data from China Health and Nutrition Survey (CHNS) spanning from 2004 to 2015, the analytical sample includes 3054 participants (aged 11–19, 48% females). We exploit ordinary least squares (OLS) and two-stage least squares (2SLS) models, obtained causal effect of Internet use on body mass index (BMI), overweight and obesity among adolescents. We find a significant positive effect of Internet use on BMI (*β* = 0.499, *p* < 0.05) and probability of overweight (*β* = 0.061, *p* < 0.05). Specifically, Internet use increases BMI by 2.56%, and increases the probability of being overweight by 6.1%. But no evidence shows that Internet use significantly increases the probability of obesity (*p* > 0.1). The mechanism is that Internet use increases sedentary activities (mainly screen time) and food consumption behavior, which results in an imbalance between energy intake and expenditure that in turn increases BMI and overweight. Furthermore, the longer the time spent playing games online, the greater the probability of adolescents being overweight (*β* = 0.012, *p* < 0.1), whereas time spent watching movies online, surfing online, and chatting online is not significantly associated with body weight. Heterogeneity analyses show that the adverse effects of Internet use on adolescents’ body weight is more pronounced in rural areas and among the male group. This study suggests that reducing time spent playing games online, and increasing physical activity and cultivating healthy eating behaviour can reduce the risk of overweight and obesity in adolescents.

## Introduction

Adolescent overweight and obesity has become a public health issue of global concern [1]. According to Word Health Organization (WHO) data, in 1975, less than 1% of children and adolescents ages 5–19 were obese, while in 2016, more than 124 million children and adolescents (6% of girls and 8% of boys) were obese. In China, the overweight and obesity rate in children and adolescents aged 6–17 was respectively 11.1% and 7.9% for 2015–2019 [2]. Overweight and obesity is the main risk factor for non-communicable diseases such as cardiovascular diseases, diabetes, musculoskeletal disorders, and some cancers (including gallbladder, kidney, and colon). Mortality associated with non-communicable diseases (NCDs) due to overweight and obesity increased from 5.7% in 1990 to 11.1% in 2019 [2]. The World Health Organization finds that overweight and obesity result in at least 2.8 million deaths and 35.8 million disability-adjusted life years every year all over the world [3].

Overweight and obesity are diagnosed by measuring a person’s weight and height and calculating the body mass index (BMI). BMI is a surrogate marker for fatness and helps in the diagnosis of obesity. In China, the rapid increase of BMI, overweight and obesity coincided with transitions in terms of society, economy and environment. Information and Communication Technology (ICT) has profoundly changed the way adolescents learn, live and play. Over the past nearly two decades, Internet use rate in China has increased from 0.05% in 1997 to 50% in 2015 [4]. In 2021, the amount of China’s underage (aged 6–18) Internet users is nearly 191 million, and the Internet penetration has increased to 96.8% [5]. Meanwhile, the prevalence of overweight and obesity among Chinese adolescents is also rising quickly. So, does the Internet penetration contribute to overweight and obesity in adolescents? If so, what are the underlying mechanisms? Hence the crucial point for policy makers is to unravel the link between Internet use and Chinese adolescents’ overweight and obesity.

The relationship between Internet use and adolescents’ BMI, overweight or obesity has been examined by several studies, but there are no consistent results [6, 7]. Several studies have shown that media use significantly increased the adolescents’ risk of obesity and overweight [8, 9]. A meta-analysis showed that every additional hour spent online each day increased the odds of being overweight and obese by 8% [8]. Cha et al. [10] found that there was a significant association between longer media use time (including playing games, watching TV and using electronic devices to access the internet) and shorter sleep duration and poorer sleep quality among adolescent students. Media use also augmented the adolescents’ amount of snacking in the evening, which resulted in obesity [11]. However, media time in these studies focused on televisions and video games rather than only Internet use [8]. Additionally, several studies have declared that Internet use decreased outdoor activities among adolescents [12, 13], which reduced calorie expenditure [14]. Internet addiction has also been associated with psychological disorders, such as neuroticism, anxiety, and depression [15], which can have a serious impact on weight control and induce eating disorder syndromes [8, 14]. However, the underlying mechanisms by which Internet use may affect body weight among adolescents are unclear.

Other studies have also found that Internet use is insignificantly related to adolescent BMI or overweight/obesity [16, 17]. For example, Jackson et al. [18], based on 482 children (including 1/3 African American and 2/3 Caucasian American) with an average age of 12, found that Internet use was not significantly associated to BMI or overweight. Moreover, Internet use can lead to better literacy skills and higher self-esteem for children or adolescents [19], hence improve their health [20]. A study showed that Internet use did not augment the risk of overweight in girls [21]. It has even been found that access to the Internet reduced the incidence of overweight in individuals through health information search, preventive health service and physical activity [4]. The contradiction of the above findings may be due to the heterogeneity of the study population, participant gender of, country, and study design.

We are aware of a closely related paper by Ma et al. [13], who used obesity data of students from 19 middle and high schools in Shanghai, China. They found that there was a significant and positive association between problematic smartphone use and obesity among children and adolescents, and that this association differed significantly by gender and educational stage. However, this cross-sectional study did not find out the causal effect of Internet use on body weight, let alone explore potential causal mechanisms. And the height and weight data they used were from school records instead of onsite measurements. Similarly, based on a cross-sectional study, Tsitsika et al. [22] found a significant association between Internet use heavier and higher risk of overweight and obesity. Taken together, many related researches about the relationship between Internet use and body weight came from cross-sectional study [10, 13], urban level adults’ samples [4], or adult populations in developed countries [10, 18, 23]. Few studies have identified the causal affect of Internet use on body weight in China’s adolescents based on long-term longitudinal data.

Taking all the above analysis into account, the marginal contribution of this study is as follows: First, we use a large nationally representative sample of China Health and Nutrition Survey (CHNS) longitudinal data, rather than a small samples or cross-sectional studies. Second, we overcome the endogeneity bias by using fixed effects models and instrumental variable approach. Third, although related studies have mentioned possible mechanisms through which Internet use affected adolescent mental and physical health, such as dietary habits [8], sedentary activities [14], and sleep duration [10], the mechanisms involved are still ambiguous and have not been empirically tested. We will explore more closely the heterogeneity of effects of Internet use on BMI, overweight and obesity among adolescents.

## Theoretical frameworks and research hypotheses

We introduce the “*crowding out*” hypothesis to explore potential mechanisms between the Internet use and body weight. This hypothesis suggests that Internet use substitutes for many healthful activities, for instance exercise, sleep, and communication in person with family and friends, and thus negatively affects adolescents’ health and psychological wellbeing [24]. This suggests that Internet use not only has a direct adverse impact on adolescent weight, but also has an indirect adverse impact on adolescent weight by crowding out the time of physical activities for adolescents. Next, we specifically explore possible mechanisms by which Internet use affects body weight.

### Sedentary activities and physical activities

First, Internet use has increased sedentary activities in adolescents [8, 25], which has a close relation with body weight [26]. Individuals may meet time constraints when they are choosing which activities to participate in, and their physical activities and social activities may be replaced by the Internet use [24], which increases overweight obesity risks [23]. Some studies have shown that more screen time resulted in inactivity in adolescents [8, 27]. In a cross-sectional sample of 10–15-years-old, Sandercock et al. [28] found that higher screen time was negatively associated to lower free-time for physical activities. Meanwhile, Internet use may reduce resting metabolic rate, which is the number of calories the body burns at rest [29]. Consequently, these sedentary behaviors are related with less physical activity, lower aerobic fitness, and higher body weight and obesity among adolescents [25, 30]. Based on the above analysis, we propose hypothesis 1:

**Hypothesis 1**: Internet use increases the body weight in adolescents through sedentary activities

### Sleep duration

Second, Internet use reduces adolescent sleep duration [31], which is strongly linked to adolescent health [32]. On the one hand, a systematic review and meta-analysis suggested a strong and consistent relationship between excessive Internet use and sleep problems, shorter sleep duration, and prolonged sleep onset latency [33]. One survey showed that in 2015, more than 40% of adolescents slept less than 7 hours at night, and used electronic devices for more than 2 hours per day, which had a significant and positive association with sleep deprivation [34]. On the other hand, sleep duration plays an important role in the health of adolescents. Pathophysiologically, sleep deprivation may increase the risk of overweight and obesity by leading to more sympathetic nerve activities, higher levels of cortisol and gastrin, less leptin and diminished glucose tolerance [35]. Short sleep duration affects energy intake and energy expenditure and may result in reduced physical activity due to tiredness, altered metabolic hormones thereby increasing appetite, and individuals may alter their food choices. In addition, extra waking hours increase the consumption of energy-dense foods, all of which may form the major risk factor of obesity in adolescents [36]. Accordingly, we propose hypothesis 2:

**Hypothesis 2**: Internet use increases the body weight by crowding out sleep time

### Health behavior

As an important access to information, the Internet is frequently used by individuals to gather the health information they need [23]. Based on a sample group of adults in China, a study found that the Internet access rised the demand for individuals to purchase computers or electronics, increased the accessibility of health information, reduced risk behaviors such as cigarette and alcohol consumptions, and boosted the demand for medical services [4]. However, children and young people have a weaker ability to discern information than adults and are vulnerable to “fake news”, inaccurate and inappropriate violent and/or sexual content online. DiNardi et al. [23] suggested that Internet access increased the risk of white men and women being exposed to low quality health information, which increased obesity occurs through worsening health behaviors (e.g., smoking, alcohol use). At the same time, the Internet use has expanded individuals’ social networks, making them more suscpectible to peer effects, thus changing their health habits, such as alcohol and cigarette consumption or playing online games [37]. Therefore, we propose hypothesis 3:

**Hypothesis 3**: Internet use increases the body weight through adverse health behaviors

### Food consumption behavior

Urbanization, globalization and informatization are common trends in global development. It has been a low-budget and fast process to produce and distribute large quantities of highly processed foods [38]. In China, mobile payments, online shopping, e-commerce, and the digital economy are profoundly changing individual lifestyles, such as eating, commuting, and entertainment [39]. The Internet has also profoundly changed the food consumption behavior of adolescents, such as online food delivery services which increased calorie, salt, and fat intake [2]. In addition to, diverse food consumption advertisements are presented on the Internet, and increased exposure to food advertisements can lead to increased consumption of high-calorie foods or overall foods [40]. A study has found that increased online food consumption behavior may increase the consumption of unhealthy, high-fat, and high-sugar foods, especially cheap fast food [2], which may augment the risk of overweight or obesity [38]. Accordingly, we propose hypothesis 4:

**Hypothesis 4**: Internet use increases the body weight through food consumption behavior

## Materials and methods

### Data

In this article, we select the longitudinal data from the China Health and Nutrition Survey (CHNS), which is one of the longest and most comprehensive longitudinal health surveys in China and is still ongoing open cohort. The CHNS survey is a collaboration between the Chinese Center for Disease Control and Prevention (CCDC) and the Carolina Population Center at the University of North Carolina at Chapel Hill. This survey covered 30,000 individuals in 7200 households from 15 provinces and municipal cities from 1989 to 2015 and used a multistage, random cluster process method, which randomly selected two cities and four counties in each province. And then urban districts were randomly selected for cities by the survey and villages and towns were selected for counties. The survey defined these areas as communities, and finally selected arbitrarily households among these communities.

The CHNS contains a wealth of individual (e.g., health, nutritional and socioeconomic information, Internet use and demographic characteristics) and household-level information (household assets, sanitation, etc.). One of the main strengths of the CHNS is that weight and height are measured by medical staff rather than self-reported by the participants. This reduces the incidence of individuals misreporting their weight, as participants are likely to underreport their weight, especially heavier participants. So far, data from ten waves (1989, 1991, 1993, 1997, 2000, 2004, 2006, 2009, 2011 and 2015) are available. The CHNS questionnaire used in this article has good reliability and validity. After calculation, the Cronbach’s alpha coefficient of the questionnaire is 0.814, and the Kaiser Meyer Olkin (KMO) is 0.836, indicating that the questionnaire has good reliability and validity. Meanwhile, Chen and Liu [4] also evaluated and demonstrated the reliability and validity of the questionnaire when exploring obesity issues using CHNS.

In this study, our analysis initially included 12484 participants aged 11–19 from surveys conducted between 1989 to 2015. Since the Internet use data was only investigated in 2004 and later, we excluded the sample before 2004 (n = 7786). Then, we excluded the following participants: height (n = 544) and weight data missing (n = 26), Internet use information missing (n = 979), education level missing (n = 91), chronic disease (n = 4) data missing or answering "don’t know". As a result, a total of 3,054 participants (1439 females and 1615 males;1950 rural and 1104 urban) were included in this analysis.

The CHNS data collection was approved by the ethical standards committee of the University of North Carolina at Chapel Hill (approval Number: 07–1963). All participants gave informed consent in China Health and Nutrition Survey. Because the present study was conducted based on the de-identified, publicly available CHNS data, it does not constitute human subject research. Its institutional review board review was waived because there was no interaction with any individual, and no identifiable private information was used.

### Measures

#### Body weight

Our dependent variable is body weight, measured by using body mass index (BMI), overweight and obesity. We select BMI which is a simple index of weight-for-height. It is widely used to categorize overweight and obesity in adolescents and is defined as a participant’ weight in kilograms divided by the square of his height in meters (kg/m^2^). Further, we define overweight (yes = 1, no = 0) and obesity (yes = 1, no = 0) according to age-and gender-specific BMI criteria provided by the Chinese Working Group on Obesity [2]. A adult is defined as overweight by WHO if his BMI> = 25, and as obese if his BMI> = 30, However, the evaluation criteria for overweight and obesity in adolescents are different from that in adults and do not apply to the above defined criteria. Therefore, we used the "*Screening for overweight and obesity among school-aged children and adolescents*" standard issued by the National Health Commission of the People’s Republic of China, which applies to screening methods for overweight and obesity among China’s children and adolescents, based on the gender-age-specific BMI screening for overweight and obesity thresholds. According to the thresholds, anyone with a BMI greater than or equal to the "overweight" cut-off point relative to gender and age group and less than the "obese" cut-off point is considered overweight, and anyone with a BMI greater than or equal to the "obese" cut-off point relative to gender and age group is considered obese. The weight and height data were recorded by professional survey enumerators. In preparation for measuring participants’ height and weight, they would ask participants to wear light clothing and remove their shoes. Using standard protocols and uniform equipment (height: SECA Stadiometer 206; weight: electronic scale), survey enumerators would directly measure participants’ height and weight to the nearest 0.1 cm and 0.1 kg, respectively.

#### Internet use

We measure the Internet use at individual level, in contrast to Chen and Liu [4] who used city-level landline telephone use rate as a proxy variable for Internet access, which would result in unobservable differences in use of Internet use at the micro-level, and we choose the independent variable from the individual micro-level, which allows us to better observe Internet use and more readily reveal the relationship between Internet use and various types of mechanistic variables. Specifically, the CHNS questionnaire asked “*can you access to the Internet*?” and a dummy variable was constructed in which the participant answered yes = 1, no = 0.

In addition to this, we further explore the effect of different types of Internet activities on body weight, and the CHNS surveyed participants on four types of Internet activities: watching movies online, surfing online, chatting online and playing games online. The survey asked participants about the average daily usage time (minutes) of each of the above Internet activities separately, as a continuous variable, which has been entered into the model to take logarithms.

#### Control variables

Refering to related study Nieto and Suhrcke [9], we choose to control variables at three levels: individual, household, and community. Individual characteristics include age, gender (male = 1, female = 0), education level, the survey asked “*what is the highest level of education you have attained*?*”* 0 = graduated from primary school, 1 = lower middle school degree, 2 = upper middle school degree, 3 = technical or vocational degree, 4 = university or college degree, 5 = master’s degree or higher, chronic disease (yes = 1, no = 0), diet knowledge, the survey asked “*choosing a diet with a lot of fresh fruits and vegetables is good for one’s health*”, 5-point Likert scale, 1 = strongly disagree, 5 = strongly agree, food preferences, the survey asked “*how much do you like fast food (KFC*, *pizza*, *hamburgers*, *etc*.*)*”, 5-point likert scale, 1 = dislike very much, 5 = like very much. Household characteristics include household size (number of people), household income (gross household income inflated to 2015). Community characteristics include community sanitation (community sanitation score, 0–10), community economic level (economic component score, 0–10), and community urbanization (community urbanization index, %).

#### Mechanism variables

Mechanism variables in this paper include sedentary activities, physical exercises, sleep duration, health behaviors and food consumption behavior. First, sedentary activities, this variable is divided into two parts, screen time and non-screen time. (1) Screen time, participants were asked about the average time (in minutes) spent watching TV, watching videos/VCDs/DVDs, and playing video games per day, and the Cronbach’s alpha between the items is calculated to be 0.814, which indicates that the reliability of the scale is good. We sum up the above items screen time to indicate the total screen time per day. (2) Non-screen time, the survey asked about the “average daily time spent reading (including books, newspapers and magazines), writing, and drawing (in minutes)”. Second, physical activities, the survey asked about the time (minutes) spent on gymnastics, martial arts (Kung Fu, etc.), dancing, track, acrobatics, field, walking, swimming, soccer, football, basketball, volleyball, tennis, badminton in every day. The calculated Cronbach’s alpha is 0.875, indicating a good internal consistency effect between these items. We sum the above items to obtain the total average daily physical activites time (minutes). Third, sleep duration, the survey asked *“how many hours each day do you usually sleep*, *including daytime and nighttime*?*”* (hours). Fourth, health behaviors, including both health risk behaviors and health-promoting behaviors. Health risk behaviors contain smoking (yes = 1, no = 0) and drinking (yes = 1, no = 0), while health-promoting behaviors include medical insurance (yes = 1, no = 0), and preventive health service. The CHNS surveyed “*during the past 4 weeks*, *did you receive any preventive health service*, *such as health examination*, *eye examination*, *blood test*, *blood pressure screening*, *tumor screening*?” (yes = 1, no = 0). Finally, food consumption behavior, the survey asked “*do you buy for yourself the kind of food or drinks you see on online commercials*?”. 5-point likert scale, 0 = very seldom (below 1 times/month), 4 = very often (above 5 times/week).

### Models

#### Ordinary least squares (OLS)

To examine the effects of Internet use on body weight among Chinese adolescents, we develop baseline regression models as follows:

$$y_{it}=\beta_{0}+\beta_{1}internet_{it}+\beta_{2}C_{it}+\gamma_{i}+\sigma_{t}+\varepsilon_{it}$$

Where *y*_*it*_ denotes the BMI, overweight and obesity for individual *i* at year *t*. *Internet* is used to denote the Internet use of participants. *C*_*it*_ denotes a set of individual-level, household-level, and community-level control variables. *β*_*0*_ is a constant term, *β*_*1*_ is the coefficient of interest that is the focus of this paper, *β*_*2*_ is the regression coefficient for a set of control variables, *γ*_*i*_ denotes individual fixed effects to control for unobservable variables that is stable over time at the individual level, such as childhood circumstances or neighborhood characteristics, and *σ*_*i*_ denotes time fixed effects, used to control for influences that do not change with the participant at the time level. *ε* is the error term clustered at the individual-year level to account for the potential correlation within each individual-year pair.

#### Two-stage least squares (2SLS) model

The main empirical challenge in determining the causality of Internet use on body weight is the potential for omitted-variable bias and reverse causality. Some individual-level unobservable factors over time can affect both Internet use and body weight, such as individual preferences and lifestyle habits over time, which are unobservable omitted variables but associated with Internet use and body weight. As another example, Internet development is often affected by many economic factors, such as the level of regional economic growth, logistics accessibility, and food prices. These confounding factors may also be important factors of weight. In addition to this, reverse causation may interfere with the causal inference of this paper as well, for example, adolescents who are already overweight or obese may be more willing to take psychological comfort in the Internet and temporarily escape from the reality of stress due to their low self-esteem and anxiety about their body size.

To effectively identify causal effects, we employ an instrumental variable approach to address the possible endogeneity problem, by using historical mobile telephone exchanges (MTX) capacity (10, 000 subscribers) at the province level as instrumental variables of Internet use. Our hypothesis is that the differences in MTX availability are exogenous to adolescent body weight, deriving from the topography characteristics of each region. MTX technology is based on the transmission of data over the user’s copper telephone line, i.e., the transmission of data over the existing voice telecommunications infrastructures. However, this distance is important to provide fast Internet, because the longer the copper line, the less bandwidth available to pass through that line. For areas with mountainous and hilly topography, it is difficult to build MTX to deliver Internet signals, so these areas are usually far away from MTX, then the band of the copper lines is not wide enough to connect quickly the Internet [41]. Most of these regions lack the infrastructures needed for MTX broadband transmission. Therefore, the level of prior regional MTX can reflect the state of regional information infrastructure construction and Internet communication capacity, which should be significantly correlated with Internet use, and the historical MTX construction is mainly to meet the needs of basic information construction at that time, which is unlikely to be associated to the current body weight. The idea of constructing this instrumental variable has been used in studies examining causal inference between the Internet and voting behavior [42], as well as that between the Internet use and fertility behavior [43, 44]. To ensure that the instruments in this paper satisfy the relevance and exclusivity constraints, we conduct a series of tests in a follow-up paper to justify the selection of instrumental variables.

Next, we propose the following 2SLS model to estimate the causal effect of Internet use on body weight:

$$IU_{it}=\alpha_{0}+\alpha_{1}MTX_{pt-1}+\alpha_{2}C_{it}+\gamma_{i}+\sigma_{t}+\varepsilon_{it}$$

$$y_{it}=\beta_{0}+\beta IU_{it}+\beta_{2}C_{it}+\gamma_{i}+\sigma_{t}+\mu_{it}$$

In the model, *IU*_*it*_ denotes the Internet use for individual *i* at date *t*, *y*_*it*_ denotes the body weight (including BMI, overweight and obesity) for individual *i* at date *t*. The instrumental variable MTX denotes the capacity of mobile telephone exchanges in province *p* in period *t-1*, and C_*it*_ denotes a set of control variables (including individual characteristics, household characteristics, and community characteristics). *γ*_*i*_ denotes individual fixed effects and *σ*_*i*_ denotes year fixed effects. *ε* and *μ* are the error term.

## Results

### Results of descriptive statistical analysis

Table 1 gives descriptive statistics for all variables. There were 3054 samples in total, the mean BMI was 19.46 (SD = 3.55). The percentages of overweight and obesity were 9.04% and 5.04%, respectively. More than half of the participants used the Internet (55.83%). The average daily time spent watching movies, surfing, chatting and playing games online was about 13, 19, 23 and 24 minutes, respectively. Among the total sample, 47.12% were females with an average age of 14.49, and their educational level were mostly in secondary (38.24%) and high school (40.93%). The proportion of participants suffering from chronic diseases was very low, at 8.32%, many participants were no smoking (95.33%) and no drinking (89.81%), 73.97% were covered by health insurance, and 95.32% were not covered by preventive health services. They meant 8.466 hours of sleep per day, averaged about 24 minutes of physical activities per day, meant about 145 minutes of screen time per day, and 95 minutes of non-screen time. In terms of diet knowledge, 64.81% of participants agreed that eating fruits and vegetables was good for health, and a higher percentage of them liked fast food (36.32%). Most of the participants purchased food seen on TV or internet less frequently than once a month (66.52%). For the family and community variables, the mean household size of the participants was 4.13 persons, the mean household income was RMB 51499.45, the mean community environmental health score was 7, the community economic level score was 6.88, and the community urbanisation index was 68.49%.

**Table 1:** Results of descriptive statistics.

	Total sample (3054)		
Variable	N (%) or mean (SD)	Variable	N (%) or mean (SD)
**BMI**	19.46(3.55)	**Smoking**	
**Overweight**		No	2468(95.33%)
No	2778(90.96%)	Yes	121(4.67%)
Yes	276(9.04%)	**Drinking**	
**Obesity**		No	2150 (89.81%)
No	2900(94.96%)	Yes	244 (10.19%)
Yes	154(5.04%)	**Medical insurance**	
**Internet use**		No	795(26.03%)
No	1349(44.17%)	Yes	2259(73.97%)
Yes	1705(55.83%)	**Preventive health service**	
**Types of Internet activities**		No	2911(95.32%)
Watching movies online	13.271(60.093)	Yes	143(4.68%)
Surfing online	19.773(60.621)	**Sleep duration**	8.466(1.11)
Chatting online	23.053(69.723)	**Physical activities**	24.082 (55.29)
Playing games online	24.963(71.136)	**Sedentary activities**	
**Gender**		Screen time	145.69(143.14)
Female	1439(47.12%)	Non screen time	95.20 (116.49)
Male	1615 (52.88%)	**Food consumption behavior**	
**Age**	14.493(2.589)	Very seldom (below 1 times/month)	2007(66.52%)
**Education level**		Seldom (1–3 times/month)	439(14.55%)
Graduated from primary school	71(2.32%)	Sometimes (1–2 times/wk)	461(15.28%)
Lower middle school degree	1168(38.24%)	Often (3–4 times/wk)	96(3.18%)
Upper middle school degree	1250(40.93%)	Very often (> 5times/wk)	14(0.46%)
Technical or vocational degree	395(12.93%)	**Household size**	4.13(1.32)
University or college degree	134(4.39%)	**Household income**	51499.45 (65860.49)
Master’s degree or higher	36(1.18%)	**Community sanitation**	7.00(2.78)
**Chronic diease**		**Community economic level**	6.88(3.13)
No	2789(91.68%)	**Community urbanization**	68.49(19.52)
Yes	253(8.32%)	**Fast food preferences**	
**Diet knowledge**		Dislike very much	108(4.81%)
Strongly disagree	25(0.98%)	Dislike	498(22.19%)
Disagree	274(10.77%)	Neutral	513(22.86%)
Neutral	380(14.94%)	Like	815(36.32%)
Agree	1648(64.81%)	Like very much	310(13.81%)
Strongly agree	216(8.49%)		

Notes: Continuous variables in Table 1 report means with standard deviations in parentheses, and categorical variables report sample sizes with percentages in parentheses.

**Fig 1:**
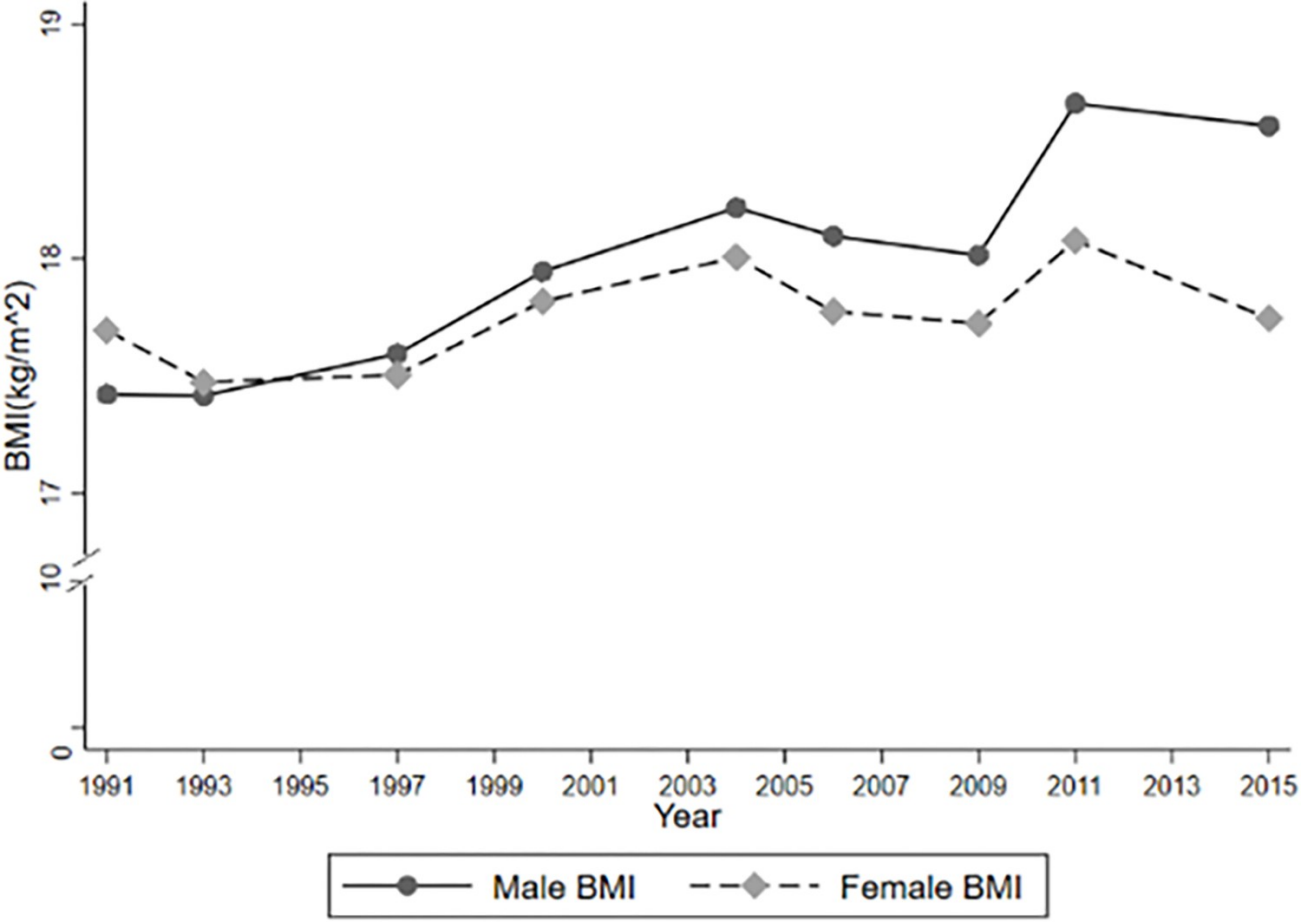
Trends of BMI during 1991–2015.

**Fig 2:**
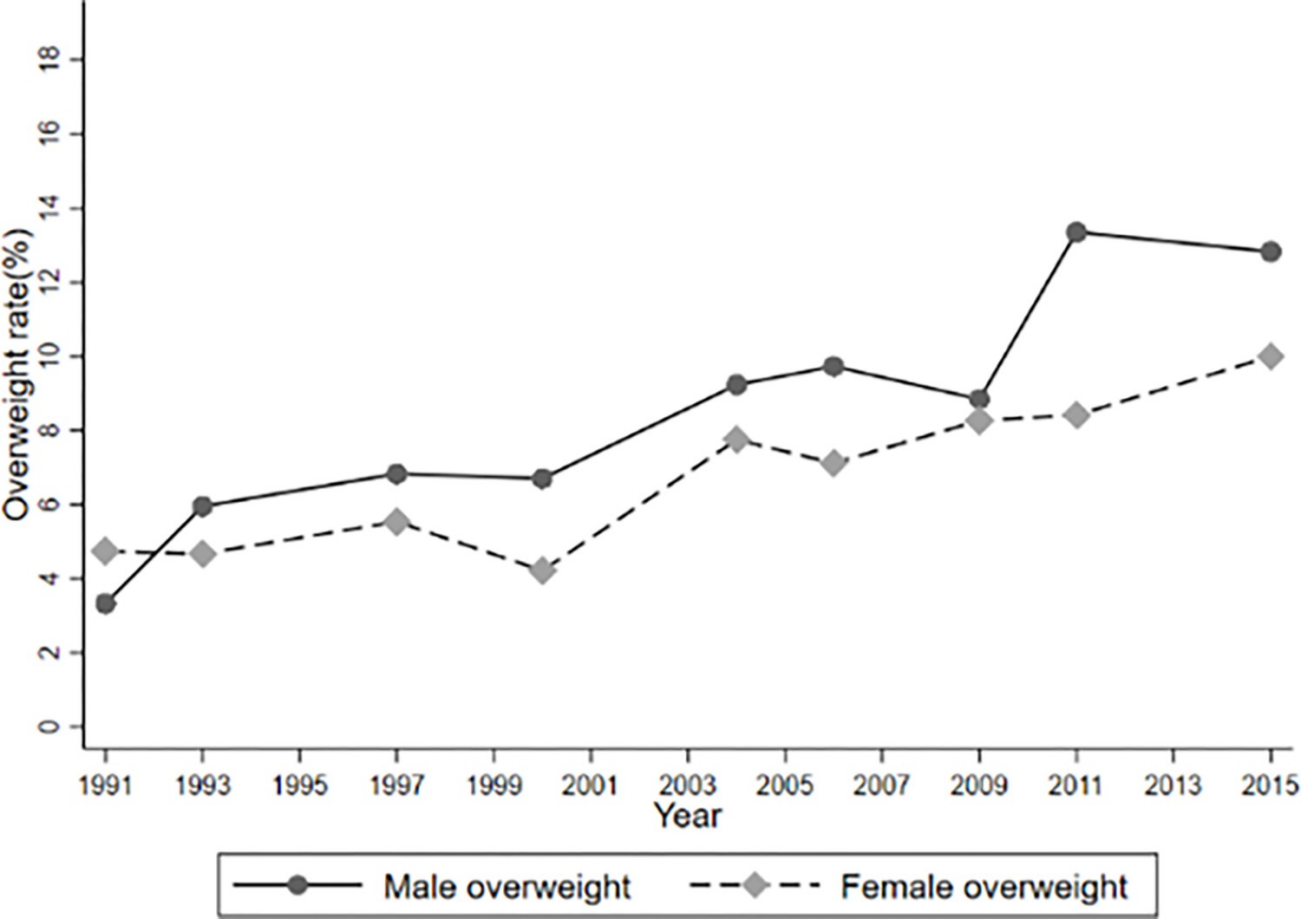
Trends of overweight during 1991–2015.

**Fig 3:**
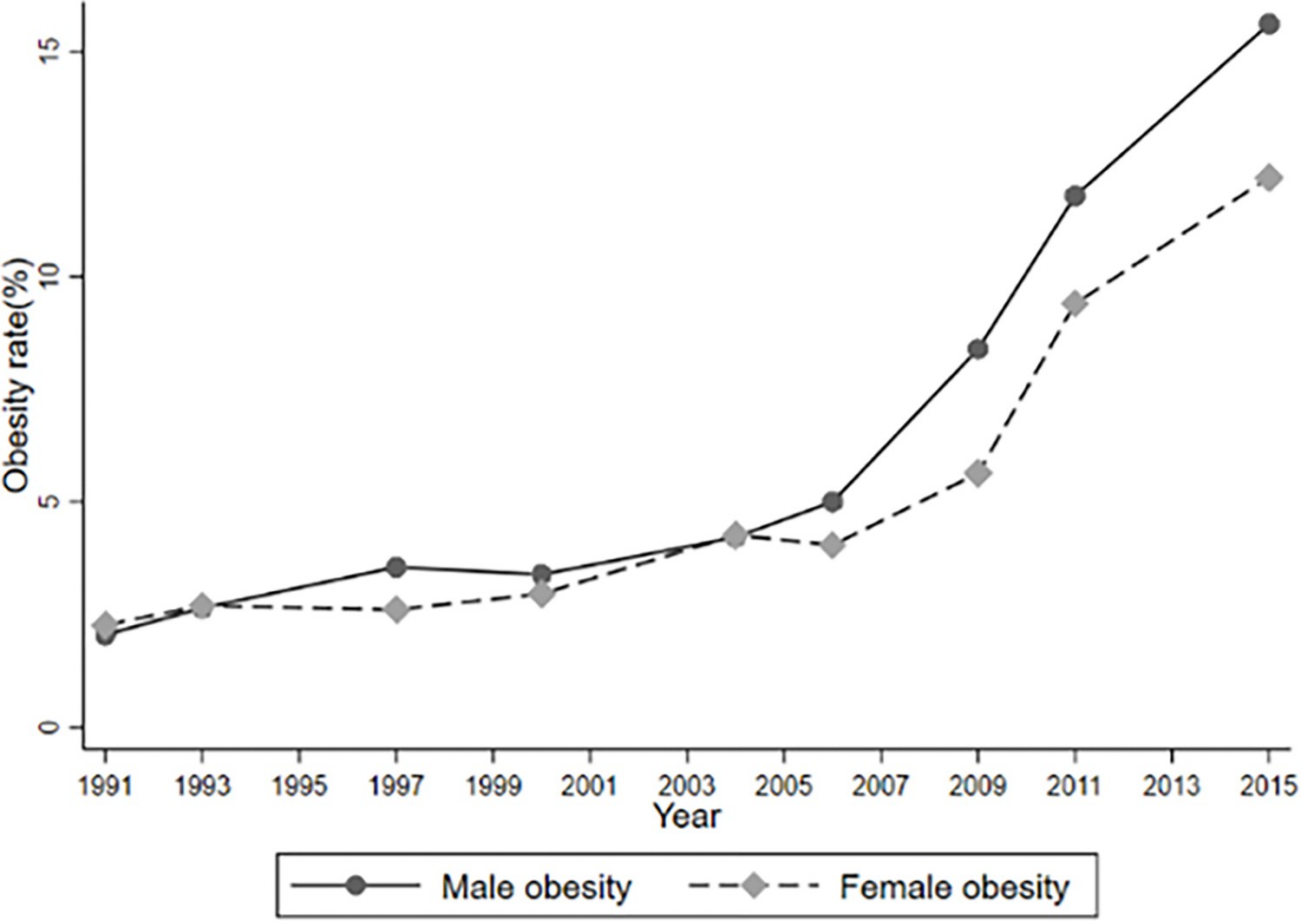
Trends of obesity during 1991–2015.

**Fig 4:**
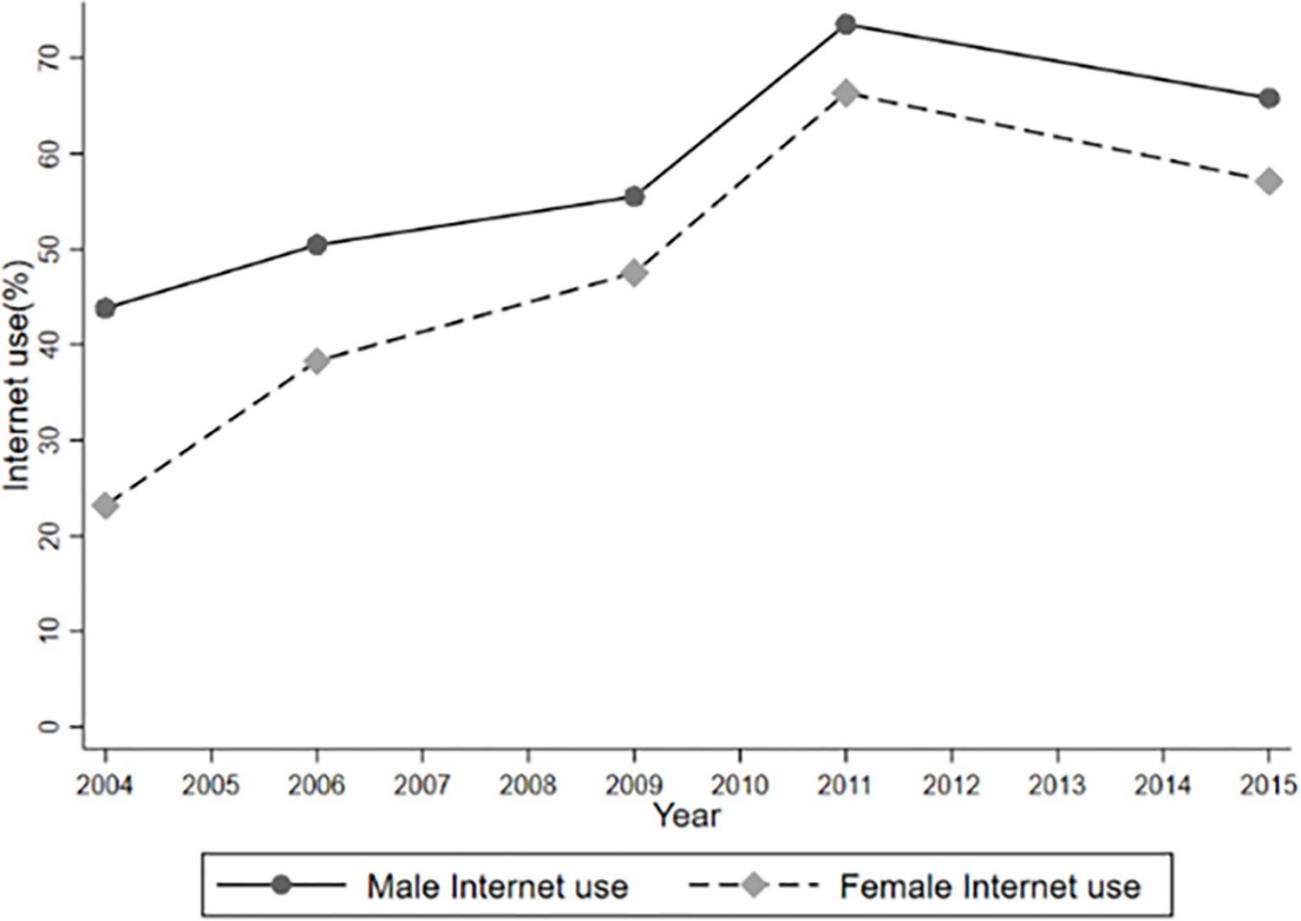
Rate of internet use during 2004–2015. Notes: Since the CHNS only started to survey individual Internet use information in 2004, the observation period for Fig 4 is 2004–2015.

In addition to, Figs 1–4 report temporal trends in Internet use and overweight among adolescents. Figs 1–3 show that the incidence of BMI, overweight and obesity among adolescents presented an upward trend during the 1991–2015 period, with obesity occurring at a faster rate, and that the BMI, obesity and overweight rates of male were generally higher than those of females (See Figs 1–3). Fig 4 also shows that the rate of Internet use grew rapidly from 2004 to 2011, with a slight decline in 2015, but the overall trend would remain upward, as the Internet use rate of minors reported by the CNNIC has already reached 96.8% in 2021.

### Baseline regression results

Table 2 shows the effect of Internet use on body weight through OLS model regression. The dependent variable in columns (1) is BMI, in (2) is overweight, and in columns (3) is obesity. First, the results show that Internet use has a significant positive effect on BMI (*β* = 0.499, *p* < 0.05). Given that the mean value of BMI in Table 1 is 19.46, Internet use increases participants’ BMI by 2.56% (0.499/19.46). Second, Internet use makes the probability of being overweight greater (*β* = 0.061, *p* < 0.05). The results denote that Internet use increases the probability of being overweight by 6.1%. In other words, participants who used the Internet had an increased risk of developing overweight compared to those who did not use the Internet. The results in columns (3) indicate that the impact of Internet use on participants’ obesity is not statistically significant.

**Table 2:** Effect of Internet use on body weight in adolescents: OLS estimation.

	BMI	Overweight	Obesity	BMI	Overweight	Obesity
	(1)	(2)	(3)	(4)	(5)	(6)
Internet use	0.499**	0.061**	-0.009			
	(0.228)	(0.028)	(0.017)			
Watching movies online				0.016	0.0002	0.0001
				(0.062)	(0.008)	(0.005)
Surfing online				-0.081	-0.001	0.002
				(0.066)	(0.008)	(0.005)
Chatting online				0.060	0.002	0.004
				(0.070)	(0.009)	(0.005)
Playing games online				0.082	0.012*	-0.004
				(0.056)	(0.007)	(0.004)
Controls variables	YES	YES	YES	YES	YES	YES
Individual fixed effects	YES	YES	YES	YES	YES	YES
Year fixed effects	YES	YES	YES	YES	YES	YES
Observations	3054	3054	3054	3054	3054	3054
R^2^	0.321	0.042	0.032	0.961	0.908	0.942

Notes: All models reported in Table 2 use OLS estimates. Control variables include individual characteristics (gender, age, education, dietary knowledge, fast food preferences), household characteristics (household size, household income) and community characteristics (community sanitation, community economic level, community urbanization). Standard errors in parentheses.

* p<0.1, ** p<0.05.

Further, the results of the OLS estimation of the four types of Internet activities with BMI, overweight and obesity are also reported in columns (4) to (6) of Table 2 for watching movies online, surfing online, chatting online, and playing games online, respectively. The results in column (4) and column (5) indicate that time spent watching movies online, playing games online, and chatting online are all positively associated with BMI and overweight, but not statistically significant. The results in column (5) show that time spent playing games online significantly increases the probability of being overweight among adolescents (*β* = 0.012, *p* < 0.1). The results in column (6) show that all four types of Internet activities are positively associated with obesity, although the estimated coefficients are small and statistically insignificant.

### IV estimates of the effect of Internet use on body weight

Although we have controlled for year fixed effects and individual fixed effects, there may still be potential endogenous problems with Internet use. For example, individual behavior or belief habits. Instrumental variable approach is used to manage the above issues. The results are revealed in Table 3. First, Columns (1), (3), and (5) show the first-stage estimates of the instrumental variables. Overall, we find that the instrumental variable MTX is positively associated to Internet use. The estimated coefficients remain stable across models and are statistically significant at the 1% level. Moreover, the first stage F-statistic is much higher than the Stock-Yogo critical value of 16.38. Second, Column (2), Column (4) and Column (6) demonstrate the impact of second-stage Internet use on BMI, overweight and obesity, respectively. The results in Column (2) denote that Internet use positively affects BMI at the 1% statistically significant level. Specifically, Internet use increases an individual’s BMI by 1.858 units (9.54%). The results in column (4) also show that Internet use significantly increases the probability of a participant’s being overweight by 30.9%. Column (6) suggests that the regression coefficient of Internet use on obesity is positive but still not statistically significant. Overall, the instrumental variable regressions are consistent with the results of the baseline regression. However, the 2SLS estimates in Table 3 are exceptionally bigger in magnitude compared to the OLS estimates in Table 2 (1.858 now versus 0.499 for the baseline, 0.309 now versus 0.061 for the baseline). This may be because the instrumental variables identify local average treatment effect (LATE), and their estimates of causal effects are only from the portion of individuals affected by the instrumental variable, which are not the average treatment effect for all participants. Despite the differences between the OLS and the IV results, we cannot reject the null hypothesis that the OLS and the IV coefficients are identical jointly. A Hausman test is conducted to examine the null hypothesis that the OLS estimates and results are identical to the IV estimates. Since the p-values from this test are 0.99 and 1, which shows there is no difference between the results of using instrumental variable regression and OLS estimation. Considering the Hausman test result, we shall rely on the OLS specification in all future specification since it is more efficient.

**Table 3:** The effect of Internet use on body weight in Chinese adolescents: 2SLS model estimation.

		BMI		Overweight		Obesity
	First-stage	Second-stage	First-stage	Second-stage	First-stage	Second-stage
	(1)	(2)	(3)	(4)	(5)	(6)
Internet use		1.858**		0.309*		0.012
		(0.767)		(0.170)		(0.072)
*MTX*_*t-1*_	0.454***		0.184***		0.289***	
	(0.075)		(0.052)		(0.062)	
F-Statics	39.37		67.17		50.46	
Control variables	Yes	Yes	Yes	Yes	Yes	Yes
Individual fixed effects	Yes	Yes	Yes	Yes	Yes	Yes
Year fixed effects	Yes	Yes	Yes	Yes	Yes	Yes
*p*-value (Hausman test)	0.999		1.000			
Observation	3054	3054	3054	3054	3054	3054

Notes: Table 3 reports the results of the 2SLS estimation. The dependent variable for columns (1), (3), and (5) is Internet use, and the dependent variables for columns (2), (4), and (6) are BMI, overweight, and obesity, respectively. The instrumental variable, mobile telephone exchange capacity (MTX) (10,000 subscribers), is from the *China Statistical Yearbook*, and we use one-year lagged MTX to measure historical infrastructure development at the provincial level, taking the natural logarithm in entering the regression model. All control variables are the same with Table 2. Standard errors in parentheses.

*** p<0.01, ** p<0.05, * p<0.1.

### Robustness checks

#### Controlling for province-specific time trends

Time fixed effects and individual fixed effects have been controlled in the baseline regressions, but over time, socio-economic characteristics of different provinces (e.g., economic level and rate of urban development across cities over time) may also affect the rate of Internet development as well as overweight and obesity, which may interfere with our estimation results. We therefore include interaction terms for the province dummy and year dummy variables in column (1) of Table 4, and the results in column (1) denote that Internet use still significantly increases participants’ BMI at the 5% statistically significant level, and the estimated coefficients are similar to the baseline regression.

**Table 4:** Results of the robustness check.

	Controlling for province-specific time trends	Including household sanitation variables	Considering migration	Winsorize	Weight	Height	Alternative instrumental variable
	(1)	(2)	(3)	(4)	(5)	(6)	(7)
Internet use	0.568**	0.549**	0.455*	0.471**	1.409*	0.052	0.551**
	(0.237)	(0.233)	(0.261)	(0.202)	(0.718)	(0.742)	(0.247)
Control variables	Yes	Yes	Yes	Yes	Yes	Yes	Yes
Individual fixed effects	Yes	Yes	Yes	Yes	Yes	Yes	Yes
Year fixed effects	Yes	Yes	Yes	Yes	Yes	Yes	Yes
Observations	3054	3054	1677	2204	3054	3054	3054
F-Statics							49.82
R^2^	0.392	0.962	0.959		0.968	0.950	

Notes: The dependent variable for columns (1), (2), (3), (4) and (7) is BMI, and the instrumental variable for column (7) is Capacity of office telephone exchanges (10,000 lines) from the *China Statistical Yearbook*. Based on the province codes, the data are matched to the CHNS using one period of lagged data. All control variables are the same with the ones in Table 2. Standard errors in parentheses.

** p<0.05, * p<0.1.

#### Including household sanitation variables

In column (2) additional control variables is added at the household level, including household drinking water quality. Household drinking water which contains in-house tap water, in-yard tap water and bottled water, takes the value of 1, which indicates good drinking water quality, and 0 otherwise. For toilet hygiene, household toilets which are indoor flushing, indoor toilets, outdoor flushing latrines, and public restroom, take the value of 1, which indicates good toilet hygiene, otherwise 0. For household sanitation, the survey asked "*is there any excreta around the dwelling place*?" as a 4-point Likert scale, with 1 = no excreta to 4 = much excreta. For clean energy, the survey asked "*what kind of fuel does your household normally use for cooking*?" and we assign a value of 1 to the use of electricity, liquefied petroleum gas, and natural gas, and a value of 0 to the use of coal, kerosene, wood, sticks/straw, and charcoal. The estimation results in column (2) are consistent with the results of the baseline regressions in column (1) of Table 2.

#### Considering migration

One concern is that Internet use may prompt population migration, which may bias our estimates. For example, parents of those economically affluent families tend to move to communities with better levels of Internet development, which somewhat increases adolescents’ Internet use and adversely affects overweight and obesity. Fortunately, the CHNS gives the move status and move-out time for all household members. The migrated sample is excluded in column (3) and the estimates remain robust.

#### Excluding BMI extremes

The descriptive statistic of Table 1 show that the mean BMI is 19.46. It is suspectable that the estimates may be affected by extreme values. Therefore, we winsorize the top and bottom 0.5% observations. After this, the results in column (4) indicate that our estimates coefficients change little.

#### Alternative measurements of body weight

In columns (5) and (6) of Table 4, the effect of Internet use on weight and height are evaluated respectively. Just as we expected, there is a significant positive effect of Internet use on weight, particularly, Internet use increases participants’ weight by 1.409 kg, or 2.41% (mean = 58.47 kg). In contrast, the effect on height is not statistically significant, which provides a placebo test for confounding. That means Internet use primarily changes an individual’s weight and has no significant effect on height.

#### Alternative instrumental variable

Based on the *China Statistical Yearbook*, the lagged one-period capacity of office telephone exchanges (OTX) is collected as an instrumental variable for Internet use. The results in column (7) denote that the F statistic is 49.82, which passes the weak instrumental variable test. The results suggest that the estimated coefficients using one-year lagged OTX as an instrumental variable are still significantly positive, further testing the robustness of the baseline regression estimates in this article.

### Mechanism tests

The second part of this paper has explored some of the potential mechanisms by which Internet use affects body weight. In column (1) and column (2) of Table 5, we regressed sedentary activities (including screen time and non-screen time) on Internet use. The result shows that Internet use affect significantly and positively screen time at the 1% statistical level. However, Internet use did not significantly increase non-screen time, suggesting that Internet use elevates adolescents’ risk of overweight or obesity primarily through increased screen time. Meanwhile, we further regressed physical activities on Internet use in column (3) which shows that Internet use has a significant positive effect on physical activities, which is an interesting finding. The follow-up discussion will explain why Internet use has increased physical activities time yet still increases body weight.

**Table 5:** Regression results of Internet use on mechanism variables.

	Sedentary activities		Physical activities	Sleep duration	Health risk behaviors		Health-promoting behaviors		Food consumption behavior
	Screen time	Non-screen time			Smoking	Drinking	Preventive health service	Medical insurance	
	(1)	(2)	(3)	(4)	(5)	(6)	(7)	(8)	(9)
Internet use	0.571***	0.405	0.664***	-0.032	0.027	0.017	-0.053	-0.043	0.255**
	(0.201)	(0.264)	(0.233)	(0.132)	(0.021)	(0.035)	(0.057)	(0.045)	(0.121)
Control variables	Yes	Yes	Yes	Yes	Yes	Yes	Yes	Yes	Yes
Individual fixed effects	Yes	Yes	Yes	Yes	Yes	Yes	Yes	Yes	Yes
Year fixed effects	Yes	Yes	Yes	Yes	Yes	Yes	Yes	Yes	Yes
Observations	3054	3054	3054	3054	3054	3054	3054	3054	3054
R^2^	0.221	0.133	0.192	0.081	0.122	0.065	0.031	0.312	0.097

Note: All models reported in Table 5 use OLS models estimates. Control variables are the same with Table 2. Standard errors in parentheses.

*** p<0.01, ** p<0.05.

Next, column (4) focuses on participants’ sleep duration, with the dependent variable being the average number of hours of sleep per day (hours), and notice that the estimated coefficient of Internet use on sleep duration is negative, but not statistically significant. This indicates that Internet use does not significantly reduce sleep duration, which can lead to weight gain.

In columns (5) to (8) of Table 5, the results of the regressions of health behaviors on Internet use are examined. The results show a positive but not statistically significant regression coefficients for Internet use on smoking and drinking. The coefficients for Internet use on preventive health service and health insurance are negative, but again not statistically significant.

The results in columns (9) of Table 5 denote that Internet use significantly increases participants’ food consumption behavior. This suggests that the popularity of the Internet and the widespread use of digital technology have spawned the rapid development of e-commerce and changed the food consumption habits and patterns of adolescents, with the growing popularity of online catering services among adolescents, and that most of this food is fast-cooked, high oil, high sugar and high salt food, which greatly increases the risk of obesity.

### Heterogeneity analysis

To test for heterogeneity in the effects of Internet use on body weight, we construct interaction terms between Internet use and participants’ age, gender, and rural areas, respectively. The results in columns (1) and (2) of Table 6 indicate that Internet use significantly has an effect positive on the BMI in rural areas compared to urban areas. Specifically, Internet use increase rural participants’ BMI by 1.780 units. In the age heterogeneity analysis, Internet use does not significantly affect participants’ BMI and overweight with age. Columns (3) to Columns (4) do not detect a significant difference in the effect by age. This may be because the adolescents in this paper are mainly distributed between the ages of 11–19, and their age span is small, with only adolescents aged 15 years and younger accounting for 64.37% of the sample, so that the effect of the Internet on body weight does not vary much across age differences. In the gender heterogeneity analysis, the results in column (5) show that Internet use significantly increases BMI in males compared to females, which is higher than the effect in the baseline regression results. This suggests that the negative effect of Internet use on males’ body weight is stronger.

**Table 6:** Results of heterogeneity analysis.

	BMI	Overweight	BMI	Overweight	BMI	Overweight
	(1)	(2)	(3)	(4)	(5)	(6)
Internet use	1.033***	0.091*	1.323	0.011	0.103	0.049
	(0.262)	(0.049)	(1.387)	(0.172)	(0.295)	(0.036)
Internet use **×** rural	1.780***	-0.042				
	(0.461)	(0.058)				
Internet use **×** age			-0.057	0.003		
			(0.094)	(0.011)		
Internet use **×** male					0.864**	0.025
					(0.413)	(0.051)
Control variables	Yes	Yes	Yes	Yes	Yes	Yes
Individual fixed effects	Yes	Yes	Yes	Yes	Yes	Yes
Year fixed effects	Yes	Yes	Yes	Yes	Yes	Yes
Observations	2204	2204	2207	2204	2207	2204
	0.348		0.322		0.329	

Notes: Heterogeneity in the effect of Internet use on obesity is no longer analysed in Table 6 as the effect of Internet use on obesity is not significant in the baseline regression results in Table 2. All models reported in Table 6 use OLS estimates. The variable *rural* is a dummy variable, rural = 1, urban = 0. All control variables are the same with the ones in Table 2 Standard errors in parentheses.

*** p<0.01

** p<0.05

* p<0.1.

## Discussion

In this paper, we explore the effect of Internet use on Chinese adolescents’ body weight using CHNS 2004–2015 longitudinal data, and notice that Internet use has a significant positive effect on adolescents’ BMI and probability of being overweight, whereas there is no evidence that Internet use significantly increases the risk of obesity. Further, increased time spent playing games online also significantly predictes adolescent overweight risk. Mechanism analyses indicate that Internet use increases adolescent body weight through increased sedentary activities (mainly screen time) and food consumption behaviour. Heterogeneity analyses showe that the adverse effects of Internet use on body weight are more pronounced in males, adolescents living in rural areas. To the best of our knowledge, this is the first study of the causal relationship between Internet use and adolescent body weight based on an instrumental variables approach using data from a nationally longitudinal survey. Also, the study in this paper analyzes the mechanisms and heterogeneity of the effects of Internet use on adolescent body weight. Next, we respond to the existing related literature based on our findings, and at the end we give some limitations of this study and future research perspectives.

### Internet use significantly increases Chinese adolescent’ BMI and overweight

We compare our evaluations with previous studies that evaluate the impact of Internet use on body weight. Chen and Liu [4] found that Internet use reduced an individual’s risk of being overweight, with each 10% increase in Internet coverage decreasing an individual’s probability of being overweight by 1.62%. Potential channels were increased personal income and preventive health behaviors (i.e., physical activity and checkups) associated with Internet use. Our findings are in contrast. Possible reasons for this are that their sample was an urban adult’s population (18 to 65 years old) and that they used only city-level Internet penetration as a proxy variable of Internet use, which ignores micro-level individual Internet use behavior. Moreover, DiNardi et al. [23] confirmed that internet access significantly increased BMI and overweight in white women, for every 10% increase in Internet coverage, white women’s BMI will increase by 0.1026 and the probability of obesity will increase by 0.006. Our study find that Internet use is more impair to males’ body weight. Specifically, compared to females, Internet use significantly increases boys’ BMI by 0.864 units. Generally, males are more active than females, especially among teenagers. A large national survey of Chinese children and adolescents reports that boys spend more time watching screens and are more unlikely to meet guidelines for limiting screen time than girls [5]. Spending more time playing computer games significantly increases the risk of overweight among adolescents, which conforms with previous related literature [45]. Moderate use of online games can increase students’ spatial imagination and cognitive skills, while addiction to online games not only impairs adolescents’ academic performance and communication skills [46], but has also a negative association with social self-esteem. Online gaming has been described as a "solitary" activity that reduces adolescents’ opportunities to interact with friends and family, which increases their sedentary activities, affects social skill development, and increases health risks as well [18, 25]. This indicates that when exploring the association between Internet use and body weight, it is also important to take full account of differences across gender, and careful consideration should be given in making specific policy recommendations. In summary, the magnitude of the effect of Internet use on BMI and overweight in Chinese adolescents is meaningful and comparable to other studies about Internet use and body weight.

### Sedentary activities and food consumption behavior are important mechanism

Our mechanism test reveals that Internet use increases sedentary activities. Interestingly, the estimates in Column (3) of Table 5 also suggest that Internet use increases physical activities, which is generally thought to reduce BMI and overweight. A possible explanation is that the data we use on physical exercises do not reflect the frequency and intensity of exercise, and that Internet use does not enable individuals to exercise at a level of intensity that results in lower body weight. Another explanation for the simultaneous increase in exercise and body weight is that participants who conduct exercise programs through Internet fitness information tend to over-estimate how many calories they burn and thus overcompensate for calories through their diet [23, 47]. This paper shows that Internet use increases participants’ time spent exercising during the previous week by 24 minutes, but lead to an increase in time spent participating in sedentary activities by 68 minutes. This again suggests that the increase in exercise due to Internet use is not sufficient to offset the calorie accumulation that occurred as a result of sedentary activity. This is consistent with epidemiologic studies that attempt to explain the phenomenon that some people exercise but lose less weight [48]. In addition to, food consumption behavior is another important channel through which the Internet increases body weight in adolescents. Internet use significantly increases adolescents’ food consumption behavior, and there is also some suggestive evidence showing that Internet use increases fat intake and decreases protein intake, although this is not statistically significant. The rapid development of Internet economy in China has given rise to the emergence of online food delivery services. The Internet use potentially increases the consumption of foods such as snacks, late night snacks and fast food (KFC), barbecue, etc., most of which are high in oil, salt, or calories, posing a potential risk to overweight or obesity [49].

### Internet use increase significantly the BMI of rural adolescents

In our study, there are also important urban-rural differences in the effect of Internet use on adolescent body weight. According to a report by the CNNIC, in 2021, the Internet penetration rate of minors (aged 6–18) in rural China was 97.3%. For those who spend an average of more than 5 hours online during holidays, rural areas are 3.9% higher than urban areas. For those who play game and watch short videos online, rural areas are 6.0% and 8.3% higher than urban areas, respectively. At the same time, in the context of urbanization, majority of rural adults have left the villages, and more than 40% of rural minors Internet users do not live with both parents, which is nearly 20% higher than that of urban areas [5]. On the one hand, this makes more rural adolescents need to use cell phones for family contact, and on the other hand, it also leads to the lack of parental supervision and restraints on Internet use, which may result in the Internet addiction, thus increasing the risk of overweight or obesity. This suggests that public policy makers need to take full account of Internet use among adolescents in rural areas when designing interventions for adolescent obesity to reduce health inequalities between urban and rural areas.

### Limitations and future research

While the study provides valuable insights into the relationship between Internet use and body weight among Chinese adolescents, several limitations need to be addressed in future research. First, due to data limitations, we cannot explore additional biological and lifestyle mechanisms (slowing down the metabolism or impairing glucose tolerance). Second, the Internet use is measured relatively singularly, and the CHNS has not yet given the intensity of Internet use, as well as the duration of Internet use under different time periods of the day. Moreover, future studies should consider the evolving landscape of Internet use and its broader socio-cultural impact on adolescent health. Addressing these limitations will strengthen the evidence base and enhance the applicability of findings for public health interventions.

## Conclusion

This paper has examined the effect of Internet use on weight among Chinese adolescents. Using data from the CHNS 2004–2015 longitudinal survey, we identified causal and potential mechanisms of Internet use and body weight based on instrumental variables and fixed-effects models, and heterogeneity of Internet use on body weight was also tested by gender, age, and region. This study suggested that Internet use significantly increased adolescent BMI and overweight. Further, increased time spent playing games online also significantly predicted adolescent overweight risk. Mechanisms analyses indicated that sedentary activities and food consumption behavior were important mechanisms by which the Internet affected adolescents’ body weight. We also found that Internet use affected negatively the body weight, especially in the boys’ group and rural area. Overall, our study suggested that intervening in adolescent Internet use was an effective strategy for reducing BMI and overweight in China. Reducing sedentary activities and improving balanced diets were also important channels for blocking the overweight risks of the Internet use.

## References

[1] Centers for Disease Control and Prevention. Childhood Overweight & Obesity [Internet]. CDC. Centers for Disease Control and Prevention. 2022[cited 2023 July 21]. Available from: https://www.cdc.gov/obesity/childhood/index.html

[2] Pan XF, Wang L, Pan A. Epidemiology and determinants of obesity in China. The lancet. 2021;9(6): 373–392. doi: 10.1016/S2213-8587(21)00045-0 34022156

[3] Word Health Organization. Obesity and overweight [Internet]. WHO. World Health Organization.2021 [cited 2023 July 21]. Available from: https://www.who.int/news-room/fact-sheets/detail/obesity-and-overweight 2022

[4] Chen L, Liu W. The effect of Internet access on body weight: Evidence from China. Journal of Health Economics. 2022; 85:102670. doi: 10.1016/j.jhealeco.2022.102670 36055079

[5] China Internet Network Information Center. National study on internet use by underage in 2021[Internet]. CNNIC. China Internet Network Information Center.2022[cited 2023 July 20]. Available from: https://www.cnnic.net.cn/n4/2022/1201/c116-10690.html

[6] Oduro MS, Katey D, Morgan AK, Peprah P. Problematic social media use and overweight/obesity: explanatory pathway analysis of 124 667 in-school adolescents in 39 high-income countries. Pediatric obesity.2023;18(11): e13073. doi: 10.1111/ijpo.13073 37691184

[7] Kim SY, Han S, Park EJ, Yoo HJ, Park D, Suh S, et al. (2020). The relationship between smartphone overuse and sleep in younger children: a prospective cohort study. Journal of clinical sleep medicine. 2020; 16(7): 1133–1139. doi: 10.5664/jcsm.8446 32248898 PMC7954067

[8] Aghasi M, Matinfar A, Golzarand M, Salari-Moghaddam A, Ebrahimpour-Koujan S. (2020). Internet Use in relation to overweight and obesity: A systematic review and meta-analysis of cross-sectional studies. Advances in nutrition. 2020;11(2):349–356. doi: 10.1093/advances/nmz073 31386144 PMC7442323

[9] Nieto A, Suhrcke M. The effect of TV viewing on children’s obesity risk and mental well-being: Evidence from the UK digital switchover. Journal of Health Economics.2021;80: 102543. doi: 10.1016/j.jhealeco.2021.102543 34710814

[10] Cha EM, Hoelscher DM, Ranjit N, Chen B, Gabriel KP, Kelder S, et al. Effect of media use on adolescent body weight. Preventing Chronic Disease.2018;15:141. doi: 10.5888/pcd15.180206 30468423 PMC6266426

[11] Ramos E, Costa A, Araújo J, Severo M, Lopes C. Effect of television viewing on food and nutrient intake among adolescents. Nutrition. 2013;29(11):1362–1367 doi: 10.1016/j.nut.2013.05.007 24103514

[12] Almaqhawi A, Albarqi M. The effects of technology use on children’s physical activity: a cross-sectional study in the Eastern province of Saudi Arabia. Journal of medicine and life. 2022;15(10):1240–1245. doi: 10.25122/jml-2022-0148 36420281 PMC9675307

[13] Ma Z, Wang J, Li J, Jia Y. The association between obesity and problematic smartphone use among school-age children and adolescents: a cross-sectional study in Shanghai. BMC Public Health.2021;21(1): 2067. doi: 10.1186/s12889-021-12124-6 34763684 PMC8581960

[14] Park S, Lee Y. Associations of body weight perception and weight control behaviors with problematic internet use among Korean adolescents. Psychiatry Research.2017; 251:275–280. doi: 10.1016/j.psychres.2017.01.095 28222311

[15] Bessiere K, Pressman S, Kiesler S, Kraut R. Effects of Internet use on health and depression: A longitudinal study. Journal of Medical Internet Research.2010;12(1): e6. doi: 10.2196/jmir.1149 20228047 PMC3234167

[16] Li G, Hou G, Yang D, Jian H, Wang W. Relationship between anxiety, depression, sex, obesity, and internet addiction in Chinese adolescents: A short-term longitudinal study. Addictive behaviors.2019; 90: 421–427. doi: 10.1016/j.addbeh.2018.12.009 30553156

[17] Melchior M, Chollet A, Fombonne E, Surkan PJ, Dray-Spira R. (2014). Internet and video game use in relation to overweight in young adults. American Journal of Health Promotion. 2014;28(5):321–324. doi: 10.4278/ajhp.121023-ARB-515 24779723

[18] Jackson LA, von Eye A, Fitzgerald HE, Witt EA, Zhao Y. Internet use, videogame playing and cell phone use as predictors of children’s body mass index (BMI), body weight, academic performance, and social and overall self-esteem. Computers in Human Behavior.2011;27(1), 599–604. doi: 10.1016/j.chb.2010.10.019

[19] Hunley SA, Evans JH, Delgado-Hachey M. Adolescent computer use and academic achievement. Adolescence.2005; 40: 307–318. 16114593

[20] Granic I, Lobel A, Engels RCME The benefits of playing video games. The American psychologist. 2014; 69 (1): 66–78. doi: 10.1037/a0034857 24295515

[21] Belanger RE, Akre C, Berchtold A, Michaud PA. A U-shaped association between intensity of Internet use and adolescent health. Pediatrics.2011; 127(2): 330–335. doi: 10.1542/peds.2010-1235 21242218

[22] Tsitsika AK, Andrie EK, Psaltopoulou T, Tzavara CK, Sergentanis TN, Ntanasis-Stathopoulos I, et al. Association between problematic internet use, socio-demographic variables and obesity among European adolescents. European journal of public health.2016;26(4): 617–622. doi: 10.1093/eurpub/ckw028 27114408

[23] DiNardi M, Guldi M, Simon D. Body weight and Internet access: evidence from the rollout of broadband providers. Journal of Population Economics.2019; 32: 877–913. doi: 10.1007/s00148-018-0709-9

[24] Moreno MA, Jelenchick LA, Koff R, Eickhoff JC, Goniu N, Davis A, et al. Associations between internet use and fitness among college students: an experience sampling approach. Journal of Interaction Science. 2013; 1:4

[25] Sun X, Zhao B, Liu J, Wang Y, Xu F, Wang Y, et al. (2021). A 3-year longitudinal study of the association of physical activity and sedentary behaviours with childhood obesity in China: The childhood obesity study in China mega-cities. Pediatric obesity. 2021;16(6): e12753. doi: 10.1111/ijpo.12753 33225582

[26] Mo Z, Wang H, Zhang B, Ding G, Popkin BM, Du S. (2022). The effects of physical activity and sedentary behaviors on overweight and obesity among boys may differ from those among girls in China: An open cohort study. The Journal of Nutrition. 2022;152 (5):1274–1282. doi: 10.1093/jn/nxab446 35018425 PMC9071318

[27] Liu J, Kim J, Colabianchi N, Ortaglia A, Pate RR. Co-varying patterns of physical activity and sedentary behaviors and their long-term maintenance among adolescents. Journal of physical activity & health.2010;7(4): 465–474. doi: 10.1123/jpah.7.4.465 20683088

[28] Sandercock GRH, Ogunleye A, Voss C. Screen time and physical activity in youth: thief of time or lifestyle choice?. Journal of physical activity & health.2012; 9(7):977–984. doi: 10.1123/jpah.9.7.977 21979868

[29] Matusitz J, McCormick J. Sedentarism: The effects of Internet use on human obesity in the United States. Social Work in Public Health.2012; 27(3): 250–269. doi: 10.1080/19371918.2011.542998 22486430

[30] Gewirtz O’Brien J, McPherson L, Miller K, Svetaz MV. Adolescent Health: Media Use. FP essentials.2021;507: 33–38. 34410095

[31] Mathew P, Krishnan R D. Impact of problematic internet use on the self esteem of adolescents in the selected school, Kerala, India. Archives of psychiatric nursing.2020; 34 (3): 122–128. doi: 10.1016/j.apnu.2020.02.008 32513461

[32] Mazur A, Caroli M, Radziewicz-Winnicki I, Nowicka P, Weghuber D, Neubauer D, et al. (2018). Reviewing and addressing the link between mass media and the increase in obesity among European children: The European Academy of Paediatrics (EAP) and The European Childhood Obesity Group (ECOG) consensus statement. Acta paediatrica. 2018; 107:568–576. doi: 10.1111/apa.14136 29164673

[33] Mei X, Zhou Q, Li X, et al. Sleep problems in excessive technology use among adolescent: a systemic review and meta-analysis. Sleep Science Practice.2018; 2: 9. doi: 10.1186/s41606-018-0028-9

[34] Twenge JM, Krizan Z, Hisler G. Decreases in self-reported sleep duration among U.S. adolescents 2009–2015 and association with new media screen time. Sleep medicine.2017;39:47–53. doi: 10.1016/j.sleep.2017.08.013 29157587

[35] Eisenmann J, Ekkekakis P, Holmes M. Sleep duration and overweight among Australian children and adolescents. Acta Paediatrica.2006; 95(8): 956–963. doi: 10.1080/08035250600731965 16882569

[36] Taheri S. The link between short sleep duration and obesity: we should recommend more sleep to prevent obesity. Archives of disease in childhood.2006; 91(11):881–884. doi: 10.1136/adc.2005.093013 17056861 PMC2082964

[37] Carrell SE, Hoekstra M, West JE. Is poor fitness contagious? Evidence from randomly assigned friends. Journal of Public Economics.2011;95(7): 657–663. doi: 10.1016/j.jpubeco.2010.12.005

[38] Baker EH. Overweight and obesity: Prevention and weight management. International Encyclopedia of Public Health.2017:383–389. doi: 10.1016/B978-0-12-803678-5.00309-X

[39] Wang O, Somogyi S. Consumer adoption of online food shopping in China. British Food Journal.2018; 120(12): 2868–2884. doi: 10.1108/BFJ-03-2018-0139

[40] Buijzen M, Schuurman J, Bomhof E. Associations between children’s television advertising exposure and their food consumption patterns: a household diary-survey study. Appetite.2008; 50(2–3): 231–239. doi: 10.1016/j.appet.2007.07.006 17804119

[41] Sabatini F, Sarracino F. Online Networks and Subjective Well-Being. Kyklos.2017; 70(3):456–480. doi: 10.1111/kykl.12145

[42] Falck O, Gold R, Heblich S. E-lections: Voting behavior and the Internet. American Economic Review.2014;104 (7): 2238–2265. doi: 10.1257/aer.104.7.2238

[43] Nie P, Peng X, Luo TY. Internet use and fertility behavior among reproductive-age women in China. China Economic Review.2023; 77: 101903. doi: 10.1016/j.chieco.2022.101903

[44] Billari FC, Giuntella O, Stella L. Does broadband Internet affect fertility? Population studies.2019;73(3): 297–316. doi: 10.1080/00324728.2019.1584327 30919746

[45] Haghjoo P, Siri G, Soleimani, et al. Screen time increases overweight and obesity risk among adolescents: a systematic review and dose-response meta-analysis. BMC Prim Care.2022; 23: 161. doi: 10.1186/s12875-022-01761-4 35761176 PMC9238177

[46] Subrahmanyam K, Kraut RE, Greenfield PM, Gross EF. The impact of home computer use on children’s activities and development. The Future of children.2000;10(2):123–144. 11255703

[47] Melanson EL, Keadle SK, Donnelly JE, Braun B, King NA. Resistance to exercise-induced weight loss: compensatory behavioral adaptations. Medicine and science in sports and exercise.2013; 45(8): 1600–1609. doi: 10.1249/MSS.0b013e31828ba942 23470300 PMC3696411

[48] Pontzer H. Exercise is essential for health but a poor tool for weight loss: a reply to Allison and colleagues. International Journal of Obesity.2023; 47: 98–99. doi: 10.1038/s41366-022-01248-3 36526732

[49] Maimaiti M, Zhao X, Jia M, Ru Y, Zhu S. How we eat determines what we become: opportunities and challenges brought by food delivery industry in a changing world in China. European Journal of Clinical Nutrition.2018; 72(9): 1282–1286. doi: 10.1038/s41430-018-0191-1 30185849

